# Supplementary material for: Predicting nonsense-mediated mRNA decay from splicing events in sepsis using RNA-sequencing data
Source: Life Sci Alliance. 2025 Sep 24;8(12):e202503380. doi: 10.26508/lsa.202503380 (PMC12461151; doi:10.26508/lsa.202503380)
Supplement: Supplementary file 1 [file LSA-2025-03380_TableS1.docx]

Table S1.

Percentage breakdown of each splicing event categorized as “Splicing” and “Transcription” groups in control vs sepsis (Fig. 1D).

| **Control** | | **Sepsis** | |
| --- | --- | --- | --- |
| **Splicing Events** | **Percentages (51.9%)** | **Splicing Events** | **Percentages (45%)** |
| Exon Skipping | 76.3% | Exon Skipping | 44.7% |
| Retained Intron | 9.5% | Retained Intron | 19.5% |
| Alternative Donor | 8.1% | Alternative Donor | 18% |
| Alternative Acceptor | 6.1% | Alternative Acceptor | 17.8% |
| **Transcription** | **Percentages (48.1%)** | **Transcription** | **Percentages (55%)** |
| Transcription Start | 31.7% | Transcription Start | 46.7% |
| Transcription End | 62.8% | Transcription End | 48.9% |
| Alternative First | 3.9% | Alternative First | 3% |
| Alternative Last | 1.6% | Alternative Last | 1.4% |
