# Supplementary material for: Predicting nonsense-mediated mRNA decay from splicing events in sepsis using RNA-sequencing data
Source: Life Sci Alliance. 2025 Sep 24;8(12):e202503380. doi: 10.26508/lsa.202503380 (PMC12461151; doi:10.26508/lsa.202503380)
Supplement: Supplementary file 2 [file LSA-2025-03380_TableS2.docx]

Table S2.

Frequency (in percentage) of each splicing event subtype in control vs sepsis (Fig. 1E).

| **Splicing Events** | **Control** | **Sepsis** | **p value** |
| --- | --- | --- | --- |
| Exon Skipping | 76.3% | 44.7% | < 0.001 |
| Retained Intron | 9.5% | 19.5% | < 0.001 |
| Alternative Acceptor | 8.1% | 18% | < 0.001 |
| Alternative Donor | 6.1% | 17.8% | < 0.001 |
