# Supplementary material for: Predicting nonsense-mediated mRNA decay from splicing events in sepsis using RNA-sequencing data
Source: Life Sci Alliance. 2025 Sep 24;8(12):e202503380. doi: 10.26508/lsa.202503380 (PMC12461151; doi:10.26508/lsa.202503380)
Supplement: Supplementary file 3 [file LSA-2025-03380_TableS3.docx]

Table S3.

Percentage breakdown of each splicing event categorized as “Splicing” and “Transcription” groups in control vs sepsis (Fig. 1H).

| **Control** | | **Sepsis** | |
| --- | --- | --- | --- |
| **Splicing Events** | **Percentages (50.5%)** | **Splicing Events** | **Percentages (37.9%)** |
| Exon Skipping | 76.1% | Exon Skipping | 48.5% |
| Retained Intron | 9.5% | Retained Intron | 21.7% |
| Alternative Donor | 8.2% | Alternative Donor | 15.6% |
| Alternative Acceptor | 6.2% | Alternative Acceptor | 14.2% |
| **Transcription** | **Percentages (49.5%)** | **Transcription** | **Percentages (62.1%)** |
| Transcription Start | 33.6% | Transcription Start | 51% |
| Transcription End | 61% | Transcription End | 46% |
| Alternative First | 1.6% | Alternative First | 1.8% |
| Alternative Last | 3.8% | Alternative Last | 1.2% |
