# Supplementary material for: Predicting nonsense-mediated mRNA decay from splicing events in sepsis using RNA-sequencing data
Source: Life Sci Alliance. 2025 Sep 24;8(12):e202503380. doi: 10.26508/lsa.202503380 (PMC12461151; doi:10.26508/lsa.202503380)
Supplement: Supplementary file 4 [file LSA-2025-03380_TableS4.docx]

Table S4.

Frequency (in percentage) of each splicing event subtype in survived vs deceased (Fig. 1I).

| **Splicing Events** | **Survived** | **Deceased** | **p value** |
| --- | --- | --- | --- |
| Exon Skipping | 76.1% | 48.5% | < 0.001 |
| Retained Intron | 9.5% | 21.7% | < 0.001 |
| Alternative Acceptor | 8.2% | 15.6% | < 0.001 |
| Alternative Donor | 6.2% | 14.2% | < 0.001 |
