# Supplementary material for: Predicting nonsense-mediated mRNA decay from splicing events in sepsis using RNA-sequencing data
Source: Life Sci Alliance. 2025 Sep 24;8(12):e202503380. doi: 10.26508/lsa.202503380 (PMC12461151; doi:10.26508/lsa.202503380)
Supplement: Supplementary file 5 [file LSA-2025-03380_TableS5.docx]

Table S5.

Percentage of splicing events predicted to induce NMD in control vs sepsis (top) and the percentage of predicted NMD stratified by splicing subtypes (bottom) (Fig. 2B). Total Canonical refers to ENSEMBL canonical transcripts that NMD pipeline is built to process.

| **Percentage of NMD in Control vs Sepsis groups** | | | |
| --- | --- | --- | --- |
|  | Control | Sepsis | p value |
| Total Splicing Events | 114,487 | 994 |  |
| Total Canonical | 95,430 | 676 | -- |
| Predicted NMD True | 86,581 (90.7%) | 631 (93.3%) | 0.03 |
| Predicted NMD False | 8,849 (9.3%) | 45 (6.7%) | -- |
| **Percentage of NMD in Control vs Sepsis groups per subtype** | | | |
|  | NMD True in Control | NMD True in Sepsis | p value |
| Exon Skipping | 69,732 (89.7%) | 245 (92.4%) | 0.16 |
| Retained Intron | 9,272 (92.4%) | 168 (97%) | 0.61 |
| Alternative Acceptor | 3,750 (92.8%) | 102 (95.3%) | 0.43 |
| Alternative Donor | 3,827 (94.4%) | 107 (89.2%) | 0.03 |
