# Supplementary material for: Predicting nonsense-mediated mRNA decay from splicing events in sepsis using RNA-sequencing data
Source: Life Sci Alliance. 2025 Sep 24;8(12):e202503380. doi: 10.26508/lsa.202503380 (PMC12461151; doi:10.26508/lsa.202503380)
Supplement: Supplementary file 6 [file LSA-2025-03380_TableS6.docx]

Table S6.

Proportion of splicing events of transcripts predicted to cause NMD per each splicing subtype in control vs sepsis (Fig. 2C).

| **Splicing Events** | **Control** | **Sepsis** | **p value** |
| --- | --- | --- | --- |
| Exon Skipping | 80.5% | 39.4 % | < 0.001 |
| Retained Intron | 10.7% | 27% | < 0.001 |
| Alternative Acceptor | 4.3% | 16.4% | < 0.001 |
| Alternative Donor | 4.4% | 17.2% | < 0.001 |
