# Supplementary material for: Predicting nonsense-mediated mRNA decay from splicing events in sepsis using RNA-sequencing data
Source: Life Sci Alliance. 2025 Sep 24;8(12):e202503380. doi: 10.26508/lsa.202503380 (PMC12461151; doi:10.26508/lsa.202503380)
Supplement: Supplementary file 7 [file LSA-2025-03380_TableS7.docx]

Table S7.

Total and median number of premature termination codons (PTCs) generated per splicing subtype in control vs sepsis (Fig. 2D).

|  | **Control** | | | **Sepsis** | | |  |
| --- | --- | --- | --- | --- | --- | --- | --- |
|  | Total PTCs generated  per subtype | Total Events per subtype | Median PTCs per Subtype | Total PTCs generated per subtype | Total Events per Subtype | Median PTCs per Subtype | p value |
| ES | 3,873,406 | 69,732 | 37 | 17,950 | 245 | 36 | 0.51 |
| RI | 355,273 | 9,272 | 23 | 6,416 | 168 | 21 | 0.68 |
| AA | 150,304 | 3,750 | 24 | 3,738 | 102 | 24 | 0.74 |
| AD | 174,121 | 3,827 | 30 | 5,029 | 107 | 35 | 0.43 |
