# Supplementary material for: Predicting nonsense-mediated mRNA decay from splicing events in sepsis using RNA-sequencing data
Source: Life Sci Alliance. 2025 Sep 24;8(12):e202503380. doi: 10.26508/lsa.202503380 (PMC12461151; doi:10.26508/lsa.202503380)
Supplement: Supplementary file 8 [file LSA-2025-03380_TableS8.docx]

Table S8.

Percentage of splicing events predicted to induce NMD in survived vs deceased (top) and the percentage of predicted NMD stratified by splicing subtypes (bottom) (Fig. 2E). Total Canonical refers to ENSEMBL canonical transcripts that NMD pipeline is built to process.

| **Percentage of NMD in Survived versus Deceased groups** | | | |
| --- | --- | --- | --- |
|  | Survived | Deceased | p value |
| Total Splicing Events | 118,152 | 866 | -- |
| Total Canonical | 98,177 | 579 | -- |
| Predicted NMD True | 89,095 (90.8%) | 540 (93.3%) | 0.04 |
| Predicted NMD False | 9,082 (9.3%) | 39 (6.7%) | -- |
| **Percentage of NMD in Survived versus Deceased groups per subtype** | | | |
|  | Survived | Deceased | p value |
| Exon Skipping | 71,698 (89.7%) | 216 (89.6%) | 1 |
| Retained Intron | 9,495 (96.9%) | 167 (96%) | 0.64 |
| Alternative Acceptor | 3,856 (92.7%) | 75 (94.9%) | 0.59 |
| Alternative Donor | 4,046 (94.4%) | 82 (96.5%) | 0.56 |
