# Supplementary material for: Predicting nonsense-mediated mRNA decay from splicing events in sepsis using RNA-sequencing data
Source: Life Sci Alliance. 2025 Sep 24;8(12):e202503380. doi: 10.26508/lsa.202503380 (PMC12461151; doi:10.26508/lsa.202503380)
Supplement: Supplementary file 9 [file LSA-2025-03380_TableS9.docx]

Table S9.

Proportion of splicing events of transcripts predicted to cause NMD per each splicing subtype in survived vs deceased (Fig. 2F).

| **Splicing Events** | **Survived** | **Deceased** | **p value** |
| --- | --- | --- | --- |
| Exon Skipping | 80.5% | 40 % | < 0.001 |
| Retained Intron | 10.7% | 30.9% | < 0.001 |
| Alternative Acceptor | 4.3% | 13.9% | < 0.001 |
| Alternative Donor | 4.5% | 15.2% | < 0.001 |
