# Supplementary material for: Predicting nonsense-mediated mRNA decay from splicing events in sepsis using RNA-sequencing data
Source: Life Sci Alliance. 2025 Sep 24;8(12):e202503380. doi: 10.26508/lsa.202503380 (PMC12461151; doi:10.26508/lsa.202503380)
Supplement: Supplementary file 10 [file LSA-2025-03380_TableS10.docx]

Table S10.

Total and median number of premature termination codons (PTCs) generated per splicing subtype in survived vs deceased (Fig. 2G).

|  | **Survived** | | | **Deceased** | | |  |
| --- | --- | --- | --- | --- | --- | --- | --- |
|  | Total PTCs generated  per subtype | Total Events per subtype | Median PTCs per Subtype | Total PTCs generated per subtype | Total Events per Subtype | Median PTCs per Subtype | p value |
| ES | 3,985,343 | 71,698 | 37 | 10,793 | 216 | 39.5 | 0.73 |
| RI | 362,735 | 9,495 | 23 | 5,070 | 167 | 19 | 0.07 |
| AA | 154,298 | 3,856 | 24 | 2,580 | 75 | 18 | 0.47 |
| AD | 186,108 | 4,046 | 31 | 3,472 | 82 | 28.5 | 0.58 |
