# Supplementary material for: Predicting nonsense-mediated mRNA decay from splicing events in sepsis using RNA-sequencing data
Source: Life Sci Alliance. 2025 Sep 24;8(12):e202503380. doi: 10.26508/lsa.202503380 (PMC12461151; doi:10.26508/lsa.202503380)
Supplement: Supplementary file 11 [file LSA-2025-03380_TableS11.docx]

Table S11.

GO Enrichment Analysis results for all transcripts with splicing events not expected to undergo NMD with p < 0.01 in control vs sepsis (Fig. 2H).

| ID | Description | Gene Ratio | p value | p adjust | Gene ID | Count |
| --- | --- | --- | --- | --- | --- | --- |
| GO:0010800 | positive regulation of peptidyl-threonine phosphorylation | 3/40 | 0.00003 | 0.03051 | EGF/PLK1/STOX1 | 3 |
| GO:0010799 | regulation of peptidyl-threonine phosphorylation | 3/40 | 0.00011 | 0.06024 | EGF/PLK1/STOX1 | 3 |
| GO:0010288 | response to lead ion | 2/40 | 0.00090 | 0.24087 | PPP5C/BACE1 | 2 |
| GO:0009162 | deoxyribonucleoside monophosphate metabolic process | 2/40 | 0.00108 | 0.24087 | TK1/NT5C | 2 |
| GO:0018107 | peptidyl-threonine phosphorylation | 3/40 | 0.00110 | 0.24087 | EGF/PLK1/STOX1 | 3 |
| GO:0018210 | peptidyl-threonine modification | 3/40 | 0.00146 | 0.25742 | EGF/PLK1/STOX1 | 3 |
| GO:1905332 | positive regulation of morphogenesis of an epithelium | 2/40 | 0.00263 | 0.25742 | EGF/STOX1 | 2 |
| GO:0007143 | female meiotic nuclear division | 2/40 | 0.00308 | 0.25742 | PLK1/HSF2BP | 2 |
| GO:0043124 | negative regulation of canonical NF-kappaB signal transduction | 2/40 | 0.00811 | 0.25742 | TRIM59/TSPAN6 | 2 |
| GO:1905330 | regulation of morphogenesis of an epithelium | 2/40 | 0.00836 | 0.25742 | EGF/STOX1 | 2 |
| GO:0006399 | tRNA metabolic process | 3/40 | 0.00890 | 0.25742 | GATB/EXOSC7/PUSL1 | 3 |
