# Supplementary material for: Predicting nonsense-mediated mRNA decay from splicing events in sepsis using RNA-sequencing data
Source: Life Sci Alliance. 2025 Sep 24;8(12):e202503380. doi: 10.26508/lsa.202503380 (PMC12461151; doi:10.26508/lsa.202503380)
Supplement: Supplementary file 12 [file LSA-2025-03380_TableS12.docx]

Table S12.

GO Enrichment Analysis results for all transcripts with splicing events not expected to undergo NMD with p < 0.01 in survived vs deceased (Fig. 2I).

| ID | Description | Gene Ratio | p value | p adjust | Gene ID | Count |
| --- | --- | --- | --- | --- | --- | --- |
| GO:0045471 | response to ethanol | 3/31 | 0.00104 | 0.21654 | SDF4/NQO1/RPL15 | 3 |
| GO:0009411 | response to UV | 3/31 | 0.00202 | 0.21654 | MAP4K3/SDF4/CCND1 | 3 |
| GO:0019674 | NAD metabolic process | 2/31 | 0.00493 | 0.21654 | NQO1/QPRT | 2 |
| GO:0044772 | mitotic cell cycle phase transition | 4/31 | 0.00606 | 0.21654 | ACTB/KDM8/CCND1/BIRC5 | 4 |
| GO:0046470 | phosphatidylcholine metabolic process | 2/31 | 0.00689 | 0.21654 | PEMT/PLAAT2 | 2 |
| GO:0000079 | regulation of cyclin-dependent protein serine/threonine kinase activity | 2/31 | 0.00724 | 0.21654 | ACTB/CCND1 | 2 |
| GO:1904029 | regulation of cyclin-dependent protein kinase activity | 2/31 | 0.00779 | 0.21654 | ACTB/CCND1 | 2 |
| GO:0097305 | response to alcohol | 3/31 | 0.00863 | 0.21654 | SDF4/NQO1/RPL15 | 3 |
| GO:0030071 | regulation of mitotic metaphase/anaphase transition | 2/31 | 0.00954 | 0.21654 | ACTB/BIRC5 | 2 |
